# The Evoked Compound Action Potential as a Predictor for Perception in Chronic Pain Patients: Tools for Automatic Spinal Cord Stimulator Programming and Control

**DOI:** 10.3389/fnins.2021.673998

**Published:** 2021-07-12

**Authors:** Julie G. Pilitsis, Krishnan V. Chakravarthy, Andrew J. Will, Karen C. Trutnau, Kristin N. Hageman, David A. Dinsmoor, Leonid M. Litvak

**Affiliations:** ^1^Department of Neurosurgery, Albany Medical Center, Albany, NY, United States; ^2^Department of Anesthesiology, University of California, San Diego, La Jolla, CA, United States; ^3^Twin Cities Pain Clinic, Edina, MN, United States; ^4^Medtronic PLC, Minneapolis, MN, United States

**Keywords:** evoked potential, closed-loop (CL), neuromodulation, perception, spinal cord stimulation, pain

## Abstract

**Objectives:**

Spinal cord stimulation (SCS) is a drug free treatment for chronic pain. Recent technological advances have enabled sensing of the evoked compound action potential (ECAP), a biopotential that represents neural activity elicited from SCS. The amplitudes of many SCS paradigms – both sub- and supra-threshold – are programmed relative to the patient’s perception of SCS. The objective of this study, then, is to elucidate relationships between the ECAP and perception thresholds across posture and SCS pulse width. These relationships may be used for the automatic control and perceptually referenced programming of SCS systems.

**Methods:**

ECAPs were acquired from 14 subjects across a range of postures and pulse widths with swept amplitude stimulation. Perception (PT) and discomfort (DT) thresholds were recorded. A stimulation artifact reduction scheme was employed, and growth curves were constructed from the sweeps. An estimate of the ECAP threshold (ET), was calculated from the growth curves using a novel approach. Relationships between ET, PT, and DT were assessed.

**Results:**

ETs were estimated from 112 separate growth curves. For the postures and pulse widths assessed, the ET tightly correlated with both PT (*r* = 0.93; *p* < 0.0001) and DT (*r* = 0.93; *p* < 0.0001). The median accuracy of ET as a predictor for PT across both posture and pulse width was 0.5 dB. Intra-subject, ECAP amplitudes at DT varied up to threefold across posture.

**Conclusion:**

We provide evidence that the ET varies across both different positions and varying pulse widths and suggest that this variance may be the result of postural dependence of the recording electrode-tissue spacing. ET-informed SCS holds promise as a tool for SCS parameter configuration and may offer more accuracy over alternative approaches for neural and perceptual control in closed loop SCS systems.

## Introduction

Spinal cord stimulation (SCS) – the precise, targeted delivery of electrical energy to the spinal cord for drug-free chronic pain control – has been an important tool for neurosurgeons, anesthesiologists, and pain management specialists since first clinical use in 1967 (Shealy et al., 1967). For many years, the gate control theory served as the putative mechanism of action for the analgesic effects of SCS (Melzack and Wall, 1965). Later work has employed bioinformatics and proteomics to elucidate the susceptibilities of the biochemical and molecular pathways of pain to SCS (Vallejo et al., 2016; Cedeño et al., 2020; Tilley et al., 2021).

Despite advances in understanding the mechanistic effects of SCS on nociceptive pathways, clinicians are still tasked with the practical realities of programming their patients’ SCS systems to achieve the desired clinical result (Sheldon et al., 2020). This process is typically an iterative endeavor between the patient and their provider. Electrodes on the stimulation leads are selected in relation to anatomical structures or loci of sensation, and stimulation amplitudes are generally set relative to perception of stimulation (Benyamin et al., 2014; Rigoard et al., 2015). The well-known postural dependencies on perception threshold must also be considered during programming (Olin et al., 1998). These dependencies apply whether or not perceptible SCS is the therapeutic intent; for instance, a given set of stimulation parameters may be sub-perception for one posture but not another. Historically, patients have been tasked with manually adjusting their stimulation parameters to account for postural shifts that result in over- or under-stimulation (Abejon et al., 2014). This burden has been eased in some instances with closed-loop SCS systems that automatically adapt stimulation parameters in response to postural shifts (Schultz et al., 2012; Kumar et al., 2018).

More recently, spinal evoked compound action potentials (ECAPs) have been studied as a direct measure of spinal cord activation that may be used to control closed-loop SCS systems (Russo et al., 2018). The spinal ECAP is described as a triphasic bipotential, the amplitude of which represents the extent of synchronous activation in the dorsal column axons in response to SCS (Parker et al., 2012). The morphology of the ECAP is influenced by the SCS pulse width employed (Chakravarthy et al., 2020). As the ECAP consists of the superposition of multiple fiber types firing together, changes in pulse width shifts the overall composition of the individual fiber types contributing to the ECAP (Anaya et al., 2020).

While recent work has considered interdependencies between SCS frequency, ECAP amplitude (*ECAP*_*amp*_), and paresthesia intensity (Gmel et al., 2021), the relationship between the ECAP and perception thresholds across posture and pulse width – a critical parameter for SCS programming – have yet to be studied. In this feasibility study, therefore, we report on a novel ECAP-based estimate of neural threshold that can accurately track the perception of SCS by blending a unique set of psycho- and biophysical findings into an analytical framework. Further, we hypothesize that the availability of these measures may be used for automated SCS parameter configuration and control, both in- and out-of-clinic.

## Materials and Methods

In this feasibility study, spinal ECAPs and perception thresholds were collected from clinical research subjects undergoing commercial SCS trials. The ECAP recordings were then processed to reduce residual stimulation artifact and estimate *ECAP*_*amp*_. Next, the *ECAP*_*amp*_ were plotted as a function of stimulation current on growth curves; key neurophysiologic attributes were calculated by fitting these plots to a unique closed-form expression of the growth curve. Finally, a novel measure of neural activation was calculated from the growth curves and related to the subjects’ perception of the SCS. These relationships were assessed across the SCS pulse width and the subjects’ postures. A more detailed treatment of these steps is provided below.

### Leads, Stimulating, and Recording System

A custom research system capable of both delivering balanced, biphasic stimulation and recording the ECAP elicited from the stimulation was utilized in this study. The system was configured to interface with commercially available, 8-electrode, 60 cm long percutaneous SCS leads (Model 977D260, Medtronic plc). Briefly, the system consists of an isolated, clinical-grade stimulator (Digitimer DS5) and amplifier (Digitimer D440). The ECAPs and associated stimulation artifact are digitized and stored off-line for further processing (Biopac MP160). Both the performance – pre-clinical and clinical – and design of the research system are detailed more fully elsewhere (Chakravarthy et al., 2020).

### Clinical Data Acquisition

This study was a non-significant risk feasibility trial assessing the effects of stimulation parameters, electrode choice, activity, and processing methods on ECAP estimation. All human clinical work for this single-site, multi-surgeon, US based study was approved by Western Institutional Review Board (WIRB study #1188981) and was conducted in accordance with the Declaration of Helsinki. Written informed consent from each subject was obtained.

Included in this analysis are fourteen ambulatory subjects already undergoing a commercial trial to assess the suitability of SCS as an aid in the management of chronic, intractable pain of the trunk and/or limbs, including unilateral or bilateral pain. Subjects received no specific treatment as a result of their participation in this study and consequently there was no control group. The sample size used here (*N* = 14) was consistent with, or exceeded that, used by others (*N* = 16 and *N* = 5) when assessing the spinal ECAP and perception thresholds (Parker et al., 2012; Gmel et al., 2021). Each subject had two partially overlapping, staggered leads placed near T9 and spanning about three vertebral levels; the exact lead placement was at the clinical discretion of the implanting physician and was selected to optimize paresthesia coverage. While others have reported placing leads linearly when recording spinal ECAPs (Parker et al., 2012), a partially overlapping, staggered midline placement is most consistent with contemporary lead placement practice (Kapural et al., 2015). At the end of the commercial trial and just prior to lead removal, the subject’s leads were connected to the research system. Spinal ECAPs were acquired from each subject across a selection of postures (seated, supine, right and/or left lateral recumbency, standing) and stimulation pulse widths (60, 90, and 120 μs) at a common frequency of 50 Hz. The stimulation frequency was fixed to avoid introducing frequency variability as a confounder on the perception and ECAP measures acquired in this study (Gmel et al., 2021). Given the ability of the subject to comfortably adopt certain postures, not all postures and pulse width combinations were tested with each subject.

The stimulation itself was delivered on a single lead in a tripolar (guarded cathode) configuration (Sankarasubramanian et al., 2011) at either end of the lead with bipolar recording electrodes assigned to the opposite end. The stimulation tripole location (cephalad or caudal) was selected per subject preference; in some instances, both configurations were used. For each stimulation recording, stimulation was gradually ramped up from 0 mA slowly over about a minute in 0.1 mA increments until the subject reported a perception of stimulation (the perception threshold, or PT). The stimulation was then ramped up further until the subject reported discomfort (the discomfort threshold, or DT). Here, DT was defined as the point at which the subject would not want to experience the stimulation for more than a half-minute. These ramped deliveries of SCS with associated biopotential recording are referred to as “growth curve sweeps” herein. Recording sessions were kept under 2 h to limit subject fatigue. All measurements and data analyses were performed identically between subjects; no specific randomization or investigator blinding was otherwise employed. Following data collection, the subjects’ leads were disconnected from the research system.

### Artifact Reduction and ECAP Estimation Methods

**FIGURE 1:**
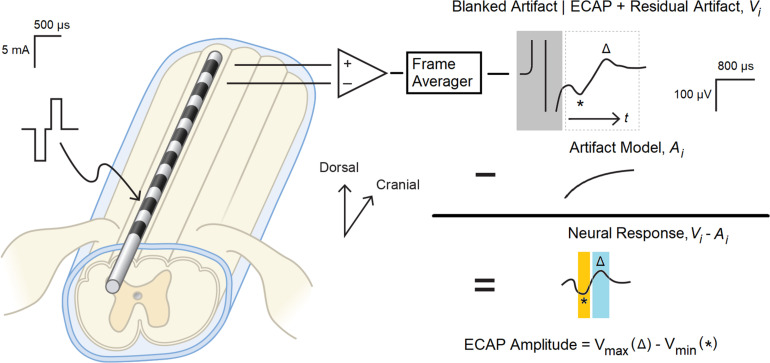
Evoked compound action potential measurement and signal processing system. Balanced, biphasic stimulation was delivered to one end of a percutaneous 1 × 8 lead, and the ECAP with associated stimulation artifact as recorded from the other end. A frame of 50 consecutive recordings was averaged (*V*_*i*_) to reduce non-synchronous noise, and the portion of the artifact coincident with the stimulation pulse (boxed in gray) was digitally blanked. A model of the underlying residual artifact (*A*_*i*_) was then calculated and subtracted from *V*_*i*_ to yield the neural response present in the biopotential recording. The ECAP was calculated by subtracting N1 (the minimum voltage in the orange window, marked with *) from P2 (the maximum voltage in the blue window, marked with △).

Artifact reduction is an important consideration for ECAP estimation, as waveforms recorded from the spinal cord may be partially corrupted by stimulation artifact (Parker et al., 2012; Chakravarthy et al., 2020). The application of an appropriate artifact reduction scheme is particularly important when assessing small amplitude ECAPs close to the perceptual threshold. Artifact reduction helps limit misclassification of non-physiologic biopotentials as “true” neural signal by the ECAP estimator. Prior to subsequent analysis, therefore, the acquired biopotentials were first averaged to reduce non-synchronous noise and then processed to reduce stimulation artifact as shown in Figure 1. All signal processing in this manuscript was performed with MATLAB (MathWorks, Natick, MA, United States).

First, consecutive frames of 50 evoked responses from each growth curve sweep were averaged (a window of 1 s, given the 20 ms period between the 50 Hz stimulation pulses) to produce a voltage waveform *V*_*i*_(*t*), with “*t*” representing time elapsed since the end of the stimulus plus 200 μs. The “*i*” in the above expression is the frame index. The 200 μs delay was chosen to blank out the artifact that manifests coincident with the delivery of the stimulation pulse (Figure 1, upper right).

After averaging, an artifact modeling method was utilized to minimize the artifact while recovering the neural response. The estimate of artifact A(t) was obtained by optimally fitting equation $A\left(t\right)=c_{1}\exp\left(\frac{t}{\tau }\right)+c_{2}t+c_{3}$ to the data V_*i*_(t) by determining parameters *c*_1_, *c*_2_, *c*_3_, τ. After the artifact model was determined, the *ECAP*_*amp*_ was then subsequently estimated as a difference (in μV) between the P2 and N1 features of the ECAP appearing in the denoised waveform *V*_*i*_ − *A*_*i*_ (Figure 1, middle right). N1 was defined as the minimum amplitude of the filtered waveform in the temporal window from 0.3 to 0.6 ms, while P2 was defined as the maximum amplitude in the temporal window from 0.7 to 1.1 ms (Figure 1, lower right). These temporal windows were set given the anticipated latencies and morphological characteristics of the ECAP (Parker et al., 2012).

### The Growth Curve and an Associated Closed-Form Expression

**FIGURE 2:**
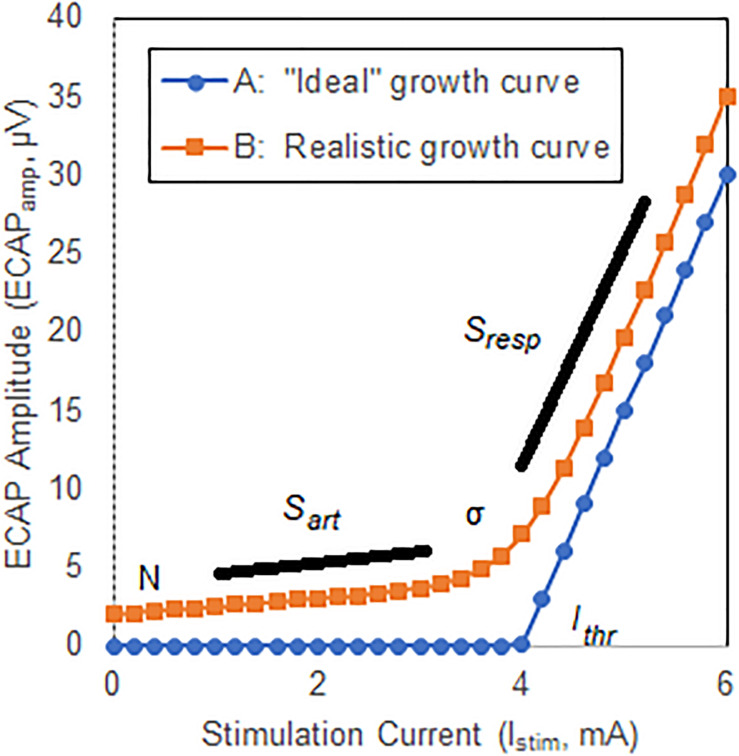
Illustrative ECAP growth curves. Curve A shows an “ideal” case where no neural activation is present up to a threshold *I*_*thr*_, at which point the neural response grows linearly with a slope *S*_*resp*_. Curve B shows a more representative model that includes curvature, σ, near neural threshold, and misclassification of stimulation artifact as neural response.

The growth curve or growth function may be defined as the relationship between the stimulation current and the estimate of neural activation as quantified with an ECAP; the threshold is defined as the intercept of the linear portion of the curve with the *x*-axis (Adenis et al., 2018). A substantially linear response is seen above threshold for growth curves acquired in the spine (Parker et al., 2012) with ideally no neural response apparent below threshold. A hypothetical example of such a growth curve is shown in Figure 2, Curve A. Here, the *ECAP*_*amp*_ is plotted as a function of the stimulation current (*I*_*stim*_). Below threshold (picked arbitrarily at 4 mA), no ECAP is observed. Above threshold, the *ECAP*_*amp*_ grows linearly at 15 μV/mA. The entire growth curve may be described completely with just two parameters: the *x*-axis intercept (*I*_*thr*_), and the slope (*S*_*resp*_) of the suprathreshold component that represents the extent of neural activation.

Two important differences exist between the ideal growth curve described above and those observed clinically, however. First, there is a substantial curvilinear component near threshold; neural activation does not instantaneously transition from zero to linear growth once a threshold is crossed. Second, a non-physiologic component of the ECAP estimate that grows linearly with increasing stimulation current – generally attributable to misclassification of stimulation artifact as ECAP – may manifest below threshold. The extent of this latter effect depends on the degree by which the signal chain rejects artifact and preserves neural response. Both these effects are shown in another hypothetical example in Figure 2, Curve B. First, a smooth transition from no neural activation to the linear response modeled with *S*_*resp*_ is introduced by means of parameter σ (described below) which is set in this example at 0.3 mA. Second, the growth curve consists of the “pure” neural activation of Figure 2, Curve A but also includes contribution from stimulation artifact misclassified as ECAP. Here, the artifact grows linearly with a slope (*S*_*art*_) of 0.5 μV/mA. An offset N of 2 μV is also included.

For analysis purposes, then, a five-parameter equation is introduced that captures the contribution *of both stimulation artifact and the underlying neural signal with associated curvature near threshold to the overall growth curve*. Such an equation is shown here:

$$ECAP_{amp}\left(I_{stim}\right)=R\left(I_{stim},I_{thr},\sigma \right)\cdot S_{Resp}+I_{stim}\cdot S_{art}+N$$

As described above, *S*_*resp*_ models the rate of growth of the response in the linear region, while *S*_*art*_ relates to rate of growth of the artifact with current. *N* captures the contribution of residual noise. The neural growth curve component *R*(*I*, *I*_*thr*_, σ) is modeled as follows:

$$R\left(I_{stim},I_{thr},\sigma \right)=\left(\sigma ln\left(exp\left(-\frac{I_{stim}-I_{thr}}{\sigma }\right)+1\right)+\left(I_{stim}-I_{thr}\right)\right)$$

The shape of *R*(*I*, *I*_*thr*_, σ) relates to the cumulative distribution of fiber thresholds in the dorsal columns, while *I*_*thr*_ and σ characterize the spreading of current between the stimulating electrodes and the dorsal column fibers. The utility of these equations lies with the potential to gain insight into the underlying neural electrophysiology and associated phenomena by analysis of the constituent components driving the morphology of the growth curve.

### Perception and the ECAP Threshold – A Novel Growth Curve Derived Measure of Neural Threshold

The ECAP threshold (ET) – a novel measure defined here for relating the ECAP to PT in the subsequent analysis – may be calculated from the expressions developed in Section “The Growth Curve and an Associated Closed-Form Expression” as:

$$ET=I_{thr}-G\sigma ,$$

with G equal to 1.5.

The basis for this equation relates to selecting a point in the neural growth curve *R*(*I*, *I*_*thr*_, σ) where (1) only a few fibers are excited [i.e., *R*(*ET*, *I*_*thr*_, σ) is close to zero], and (2) the distribution of nerve fibers $R^{\prime }\left(ET,I_{thr},\sigma \right)=\frac{1}{\left(exp\left(-\frac{ET-I_{thr}}{\sigma }\right)+1\right)}=1/\left(\exp\left(-G\right)+1\right)$ is independent of *I*_*thr*_ and σ. Regarding the first point, the perceptual threshold corresponds to excitation of only a few sensory fibers in many neural systems (Delgutte, 1990; Lobarinas et al., 2013; Tinsley et al., 2016). The second point is motivated by the observation that the current from the electrodes travels between the stimulation electrodes and fibers in the dorsal columns through, and is shunted by, the CSF (Anaya et al., 2020). The CSF thickness is dependent on anatomy of the patient as well as patient’s posture. Conceptually, the thicker the CSF, the smaller the proportion of current that is coupled into the dorsal columns. Thus, one may reasonably assume parameters *I*_*thr*_ and σ are dependent on patient posture and anatomy. By selecting ET where *R*′(*ET*, *I*_*thr*_, σ) becomes independent of these parameters, a point on the growth curve may be selected where underlying excitation of the dorsal columns is constant. While the above considerations are satisfied for any G ≫ 1, the optimal value for G (set to 1.5 in this analysis) was selected by sweeping this parameter and finding the value that maximizes the match between the ET and the psychophysical data.

Similar consideration is also relevant to the electrodes allocated for sensing. While emphasis in the literature is generally on spacing variation between the stimulating electrodes and the cord (Parker et al., 2012), variation between the sensing electrodes and the cord must be considered too. The above approach serves conceptually to desensitize the sensing electrodes as well to the anticipated posture and anatomical variation.

In this paper, growth curves from the clinically acquired sweeps – following denoising and artifact reduction – were fit to Eq. (1) by optimally adjusting parameters *I*_*thr*_, σ, *S*_*art*_, *S*_*resp*_ and *N*. ETs were then calculated from these growth curves using Eq. (3) with G = 1.5. The relationships between ET, PT, and DT across posture and pulse width were plotted.

## Results

**FIGURE 3:**
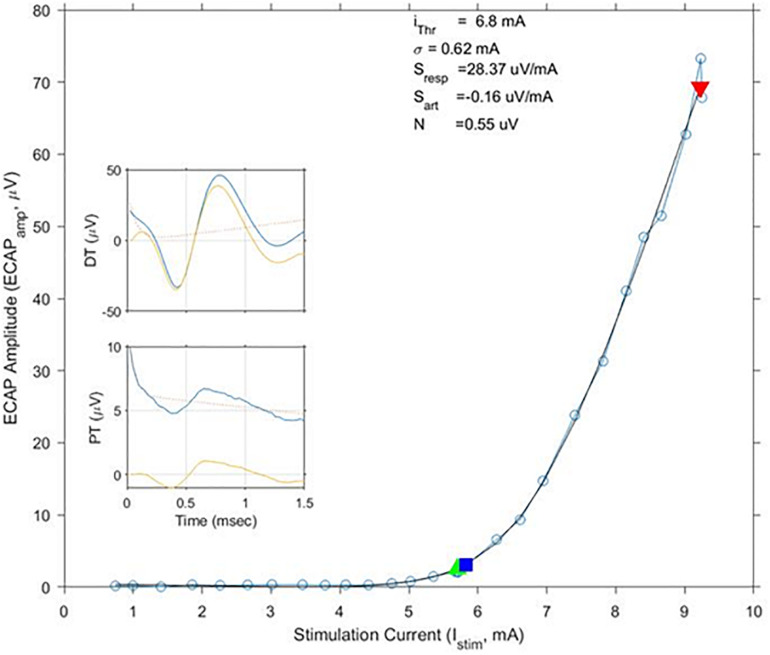
Exemplary growth curves, fit parameters, and individual ECAP recordings. This figure shows a calculated growth curve measured from subject S10 while standing. The stimulation pulse width was 120 μs. The black line shows the best matching fit of Eq. (1); fit parameters are shown above the curve. The green and red triangles indicate stimulation levels where the subjects reported PT and DT, respectively. The blue square indicates calculated ET. In this example, ET and PT closely match each other. ECAP recordings at PT and DT – both pre (blue)- and post (yellow)-artifact rejection are shown in the insets.

**FIGURE 4:**
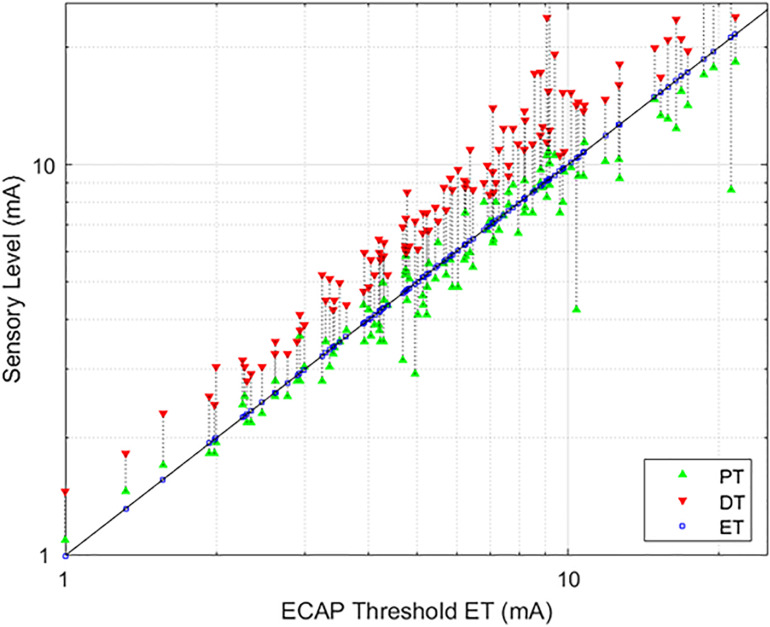
Relationships between PT and DT as a function of ET. The stimulation currents resulting in PT (green, upwards triangle) and DT (red, downwards triangle) are plotted with respect to ET; ET is also marked with a blue square for ease of reference. A green triangle without a corresponding red triangle indicates cases where DT could not be measured. In these circumstances, the required current was in excess of the maximum stimulation setting (25 mA) of the research system.

**FIGURE 5:**
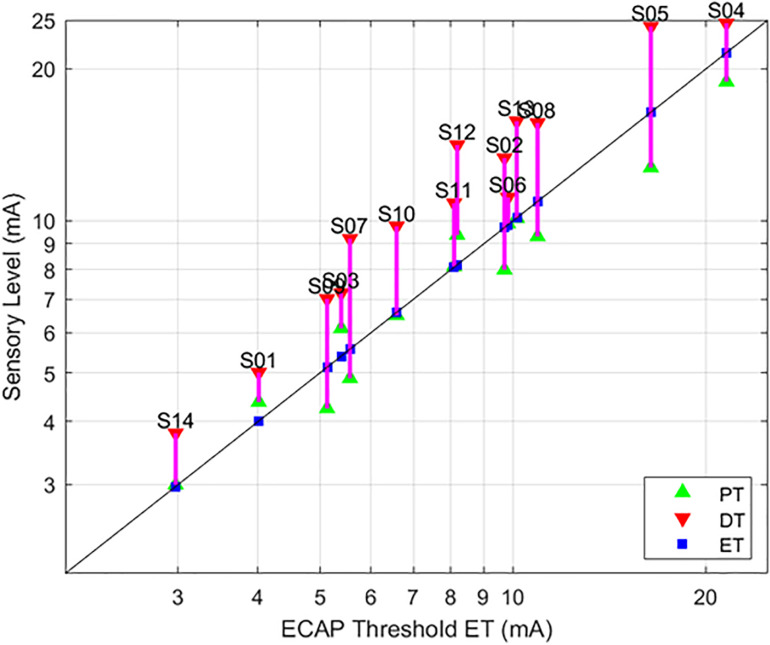
Relationships between PT and DT as a function of ET, with 90 μs stimulation while seated. This figure – incorporating a subset of data from Figure 4 – serves to illustrate the tight correlation between ET and the sensory measures employed here when posture and stimulation pulse width are controlled. The sensory measures were averaged if the subject was tested in the same condition multiple times.

**FIGURE 6:**
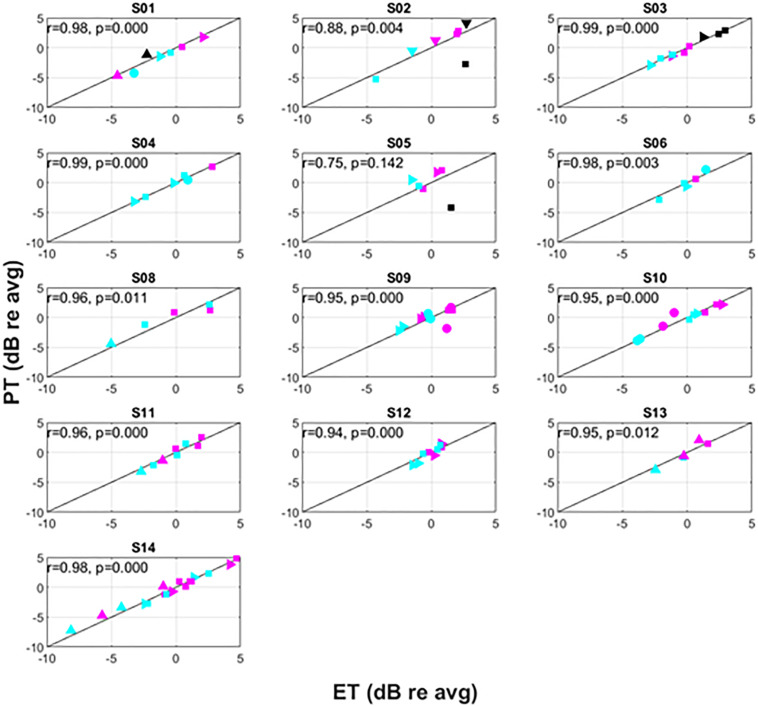
Intra-subject variation of PT versus ET. The symbols correspond to different postures as follows: supine (△), prone (▽), right lateral recumbency (⊳), sitting (□), standing (○). The colors correspond to pulse widths as follows: 60 μs (black), 90 μs (magenta), 120 μs (cyan).

**FIGURE 7:**
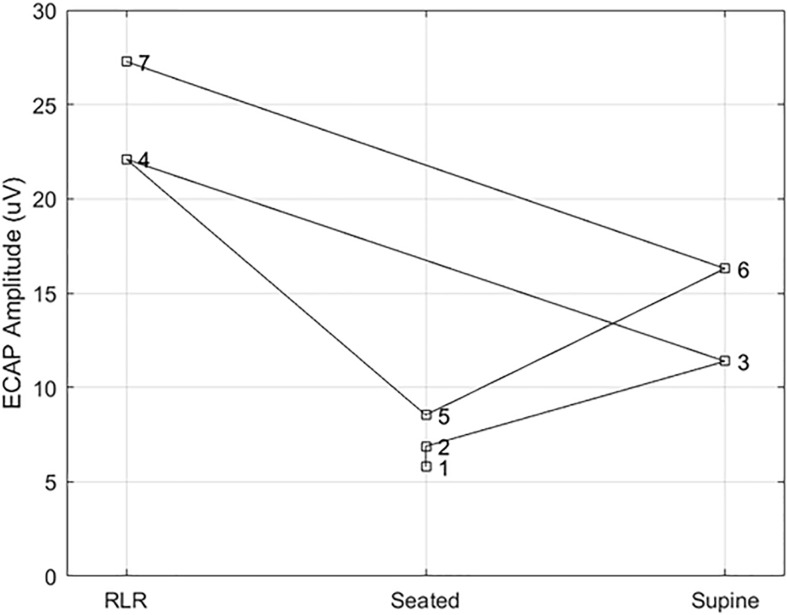
Example ECAP variability at a fixed point of perception across posture. Here, the ECAP amplitude at a fixed point of perception – in this case, the discomfort threshold – was assessed in subject S14 multiple times across three different postures (right lateral recumbency, seated, and supine). A stimulation pulse width of 90 μs was used in all cases, with the number by each marker indicating the measurement order. The ECAP measurements taken at DT for a given posture are within 5 μV of each other; however, the ECAP measurement corresponding to DT varies widely from posture to posture.

The average age of the 14 subjects was 55.9 + 12.3 years old with 7 females and 7 males. A total of 113 growth curves were obtained from the subjects, and ECAP responses could be estimated in 112 cases. The fit of the growth curves to Eq. (1) was extremely strong (*r* = 0.997; *p* < 0.0001). Two examples of the fit along with the extracted parameters are shown in Figure 3. Figure 4 shows the relationship between ET, PT, and DT across all postures and pulse widths tested. Figure 5 shows a subset of the data in Figure 4 for a single test condition (90 μs stimulation pulse width while seated). The variability of each individual subject’s PT and ET is shown in Figure 6. Finally, Figure 7 shows an example of the extent of variation in *ECAP*_*amp*_ seen at a single condition (DT) across posture.

### Relating ET, PT, and DT for the Group

As evidenced in Figure 4, strong correlations were exhibited between both ET and PT (*r* = 0.93; *p* < 0.0001, *N* = 112), and ET and DT (*r* = 0.93; *p* < 0.0001, *N* = 108). Fewer data sets are included with the ET and DT comparison since DT was not obtainable in some configurations. A subset of the data from Figure 4 was plotted separately in Figure 5; namely, those data sets where stimulation was delivered with a single pulse width (90 μs) while seated. This was done to assess whether other trends manifested when posture and pulse width were controlled. These results again exhibit ET as highly predictive of both PT (*r* = 0.98; *p* < 0.0001) and DT (*r* = 0.96; *p* < 0.0001).

Recognizing that changes in both posture and pulse width result in changes to perception threshold (Cameron and Alo, 1998; Abejon et al., 2014), Figure 6 examines the utility of ET to track changes in PT within individual subjects. Since subjects may differ widely in their average PT (Figure 5), the data for each individual subject was normalized by dividing PTs obtained for a given posture/pulse width combination by the average PT obtained across all combinations. The same operation was performed on ET. Thus, Figure 6 shows the intra-subject relationships between PT and ET in decibels across posture and pulse width. Subject 7 was not included in Figure 6, as only two conditions were tested in this subject.

### Intra-Subject ET, PT, and DT Relationships

Consistent with prior literature, large changes in PT were frequently observed across the postures assessed. Among the subjects, the largest relative change was seen in subject S02; a postural change from prone to supine position resulted in a change of approximately 12 dB. The supine position was not measured in every subject due to time constraints and subject comfort. In circumstances where it was assessed, however, it was typically associated with the lowest PT. As reported previously, PTs generally increased with decreasing pulse widths, with lowest PTs associated with 120 μs and highest PTs with 60 μs.

Changes in ET closely correlated to changes in PT within subjects, with an overall correlation between relative ET and PT of 0.91 (*p* < 0.001). Except for subject S05, this correlation was significant for all subjects with r levels between 0.75 and 0.99. In addition, the correlation was higher than 0.95 for 9 of 13 subjects. When treating ET as a predictor of DT across various conditions, the ET predicted change in DT within 0.5 dB (median, 95% confidence interval 0.4–0.6 dB obtained by bootstrapping). The accuracy in predicting changes to PT due to posture was 0.5 dB (0.3–0.6 dB) and 0.5 dB (0.4–0.6 dB) for 90 and 120 μs pulse widths, respectively. In addition, even when PTs changed substantially when subjects were asked to return to a particular posture from a different one (an example of which is included in Figure 7), ET was able to closely track the changes in PT.

## Discussion

### ET and PT Across Posture and Pulse Width

Others have previously reported that posture affects the ECAP (Parker et al., 2012). However, this is the first report describing the use of the neural threshold estimate, ET, to relate the ECAP to two clinically relevant findings about perception with posture and stimulation pulse width. First, the ET may be used to both accurately track changes in PT across patients, as well as predict changes in intra-subject PT variation with postural shift. In addition, we report the novel observation that PT and ET can vary across the same nominal posture by as much as 5 dB in some subjects. Second, the ET tracks perceptual changes associated with different pulse widths. The second finding is particularly important as various pulse widths may differentially excite particular fiber populations or volumes of neural activation in the dorsal columns (Holsheimer et al., 2011); the ability to optimize pulse width setting based on ECAPs may offer an additional programming option for patients who seek best pain relief.

This variability reported above appears subject dependent, with subjects S04 and S14 exhibiting large changes in PT and ET for the same nominal posture. Conversely, subjects S03 and S10 were very consistent across posture. It is possible that the leads were still somewhat mobile since the subjects were studied at the end of their commercial SCS trial or that anatomical factors such as spinal canal width or CSF thickness played a role. Further, the postural variability reported may or may not be representative of variability observed after permanent implantation and several months of use. Postural dependencies on stimulation perception are observed even in long-term (e.g., 4 year) SCS users, however (Cameron and Alo, 1998).

### Clinical Considerations for ET and Closed-Loop SCS

The measurement of ET involves capturing the ECAP growth curve. Accurate assessment of ET hedges on an ECAP estimation scheme – particularly near the “knee” of the growth curve, where σ is calculated – that is robust against misclassification of artifact as true neural signal. In 112 out of 113 cases, the growth curve could be measured at levels below those that were uncomfortable, suggesting the practicality of measuring ECAP growth curves either in-clinic or out-of-clinic with an implanted device. Even in the present study where stimulation was increased slowly to allow the subject to report PT and DT, the median sweep time was 46 s; the measurement can be further optimized for clinical use by utilization of adaptive procedures to rapidly estimate ET (Nehmé et al., 2014).

Previous reports of spinal ECAP sensing with associated closed-loop control focused on the utility of *ECAP*_*amp*_ as a feedback control variable for SCS (Russo et al., 2018). This report proposes the alternative measure of ET as a basis for both SCS control and perception-referenced parameter configuration. The application of the ET here – versus simply *ECAP*_*amp*_ – is potentially advantageous, owing to the desensitization of the system to the growth curve variability with perception presented in this manuscript. For closed-loop SCS systems with real-time stimulation control, system operation near the perceptual threshold approximates the performance of ET as a feedback control variable without the burden of assessing ET via repeated acquisition of the growth curve. Again, however, this benefit is only realized if the system is not susceptible to misclassification of artifact as neural activation. Yet another option may be to use a posture sensor that automatically selects different *ECAP*_*amp*_ as the feedback control variable for a closed-loop SCS system.

To better illustrate the comparative benefit of ET-informed SCS configuration and control, consider Figure 7. This example shows the *ECAP*_*amp*_ at DT for subject S14 in the right lateral recumbency (RLR), seated, and supine positions. The ECAP measurements taken at DT for a given posture are within 5 μV of each other; however, the ECAP measurement corresponding to DT differed by almost a factor of 3 between the RLR and seated positions. Thus, if the stimulation amplitude was configured to produce an ECAP of 15 μV, such stimulation would be comfortable (sub-DT) in the RLR position but uncomfortable (supra-DT) in the seated position. These findings suggest that caution is warranted when using closed-loop SCS that relies on stability of the *ECAP*_*amp*_, particularly across postures. On the other hand, ET tightly tracks (*r* = 0.98; *p* < 0.0001) the almost 4 DB of variation seen for repeated PT measurement in the same posture (Figure 6 bottom panel). If the therapeutic intent is an even perception of stimulation, the ET may offer potential as a feedback control variable for a closed-loop system.

A complete treatment of the biophysical phenomena driving ECAP variability with postural change is not provided here. As discussed in Section “Perception and the ECAP Threshold – A Novel Growth Curve Derived Measure of Neural Threshold,” though, the recording electrodes are expected to change their relative position to the spinal cord much like the stimulating electrodes do across posture and motion. Accordingly, the *ECAP*_*amp*_ variability seen with postural change may not be attributable solely to variable stimulation current coupling to the neural tissue. This theoretical argument agrees with our observation that the constant *ECAP*_*amp*_ associated with comfortable stimulation in one posture may still result in uncomfortable perception in other postures.

### Limitations

Several limitations exist with this single-site feasibility study. First, lead position differed among subjects and the anatomical features relevant to perception – such as CSF thickness and spinal canal width – were not controlled between subjects. As this study occurred at the end of a commercial SCS trial, there was not an opportunity for post-trial imaging beyond x-ray. In future work, we will obtain post-procedure CT/MRI to better assess these co-variates. Second, a limited of set pulse widths were studied. This analysis relied on small amplitude ECAP detection at the edge of perception. Even with the robust stimulation artifact reduction scheme employed here, shorter pulse widths were utilized to limit opportunity for artifact misclassification by the *ECAP*_*amp*_ estimator (Chakravarthy et al., 2020). Third, all testing was performed in-clinic under controlled experimental settings; different assessments of perception may be offered by the subjects if they were at home and engaged in everyday activities. Finally, the same parameter sets were not tested in each subject; this was primarily driven by time and comfort limitations of the subjects. Despite the limitations listed above, the analysis of the 112 growth curves acquired from the 14 subjects provides valuable insight for further research into the interdependencies between ECAP measures, posture, and stimulation configuration.

## Conclusion

Evoked compound action potential s hold promise as an important electrophysiologic biosignal to optimize the programming and control of SCS systems. While further clinical study is needed to assess the potential benefit of ET-informed neural threshold estimation versus other ECAP derived measures – such as *ECAP*_*amp*_ alone – this work demonstrates that the ET is feasible to measure and tracks perception across posture and stimulation pulse width. Collectively, these observations are of importance to clinical practice with ECAP-informed SCS systems and supports automatic SCS configuration and dose control that moves beyond the present reliance on manually acquired perception thresholds.

## Data Availability Statement

The datasets presented in this article are not readily available because the datasets are the property of Medtronic plc. Requests to access the datasets should be directed to DD.

## Ethics Statement

The studies involving human participants were reviewed and approved by Western Institutional Review Board. The patients/participants provided their written informed consent to participate in this study.

## Author Contributions

JP, KC, KH, DD, and LL took part in the conception, design, analysis, and interpretation of the data. KT, AW, DD, and KH were involved in data collection. All authors have approved the submission of this manuscript.

## Conflict of Interest

JP, KC, KT, and AW are consultants for Medtronic plc. KH, DD, and LL are employees of Medtronic plc.
